# Neonatal Palliative Care as an Integral Component of the Greek National Healthcare System: Time to Act

**DOI:** 10.7759/cureus.45498

**Published:** 2023-09-18

**Authors:** Dimitra Maria Apostolidi, Nikoletta Pantelaki, Antigoni Sarantaki, Elena Dragioti, Dimitra Metallinou

**Affiliations:** 1 Department of Midwifery, School of Health and Care Sciences, University of West Attica, Athens, GRC; 2 Research Laboratory Psychology of Patients, Families & Health Professionals, University of Ioannina, Ioannina, GRC; 3 Department of Medicine and Health Sciences, Linköping University, Linkoping, SWE

**Keywords:** greece, neonatal intensive care unit, healthcare system, neonatal, palliative care

## Abstract

Neonatal palliative care aims to provide multidisciplinary support to families and neonates suffering from life-threatening or life-limiting diseases. Many countries worldwide have recognized the importance of enhancing the quality of life in critically ill neonates and thus have created and systematically implemented palliative care protocols in neonatal intensive care units (NICUs). Europe has a very low neonatal mortality rate, which has been steadily decreasing over the last 30 years. Greece in particular, a country located in Southeast Europe, reported a neonatal mortality rate of 2.29/1,000 live births in 2020. Nevertheless, neonatal palliative care facilities are scarce on a national level. In this paper, several reasons are presented to support the integration of neonatal palliative care in the Greek national healthcare system with the vision to ensure that all neonates and their families will receive in the near future the care, support, and dignity they deserve when facing life-threatening or life-limiting illnesses.

## Editorial

The neonatal period is the most critical time for a child’s survival. Neonatal palliative care is a specialty area of medicine that focuses on giving families and neonates, with life-threatening or life-limiting diseases, medical, emotional, and psychosocial support [1,2]. Europe has a very low neonatal mortality rate, which has dramatically decreased between 1990 and 2021 [3]. Specifically, the mortality rate in 1990 was estimated at 8.21/1,000 live births while in 2021 it declined to approximately 2.46. There is evidence (vital registrations) that the Republic of Moldova in 2020 and Albania in 2021 were the first countries in Europe regarding neonatal deaths, with a rate of 6.38/ and 6.58/1,000 live births, respectively. On the contrary, Sweden and Montenegro in 2021 reported the lowest rates, 1.26 and 0.71/1,000 live births, respectively. Greece, with official notifications, determined a neonatal mortality rate of 2.29/1,000 live births in 2020 [3,4].

Regarding the situation in Greece, the Ministry of Health published in 2019 the “Palliative Care Feasibility Study for Greece” with the aim to develop and implement a palliative care strategy [5]. In this essay, with respect to pediatric palliative care, only one home service was recorded in the entire country. This service was developed in 2010 by “Merimna”, a society for the care of children and families in illness and death, and is the only one to provide medical and psychosocial care to children with life-threatening illnesses. Besides, it provides holistic support to their families or relevant caregivers (e.g. teachers) throughout the entire process. Lastly, it consists of a highly trained interdisciplinary team that cooperates with several pediatric units in both the public and private sectors.

Many countries worldwide have recognized the significance of enhancing the quality of life in critically ill neonates and thus have created and systematically implemented palliative care protocols in neonatal intensive care units, including Canada [6], Australia [7], and the UK [8]. It is of the utmost importance for each country to develop national neonatal palliative care protocols, based on its medical equipment, workforce, resources, cultural aspects, legislation, and viability thresholds, always considering international recommendations, evidence-based policymaking, and evidence-based practices. Such protocols, to our knowledge, have not yet been officially developed in Greece [5]. Integrating neonatal palliative care into the Greek national healthcare system is crucial for providing comprehensive and compassionate support to neonates and their families facing life-threatening or life-limiting conditions.

Several reasons support the integration of neonatal palliative care into the Greek national healthcare system and are summarized below.

Enhance the quality of life

While the challenges and goals of neonatal palliative care may differ from those in adult palliative care, the overall aim remains the same: to alleviate suffering and maximize the quality of life for the neonate and its family [9]. By integrating these services into the national healthcare system, healthcare professionals can provide specialized neonatal care and emotional support tailored to the unique needs of each neonate by considering the management of symptoms, controlling pain, and enhancing comfort [1,2]. Neonatal palliative care teams work closely with healthcare providers to manage discomfort in neonates. This includes addressing pain, respiratory distress, feeding difficulties, and other symptoms that may cause discomfort. Various interventions, such as medication, positioning, and non-pharmacological approaches may be used to alleviate symptoms and promote comfort [2,9].

Family-centered care

The loss of a neonate can be a devastating event and cause a profound family crisis. Neonatal palliative care recognizes the critical role of families in the care of their neonates. It supports families by providing emotional, psychosocial, spiritual, and financial/economic support, facilitating open and honest communication and decision-making, assisting with practical matters, and ensuring that families are well-informed about their neonate’s condition, treatment options, and prognosis [10]. The integration of these services ensures that families receive comprehensive support from a multidisciplinary team, including physicians, midwives, nurses, social workers, psychologists, physiotherapists, and volunteers who serve the family’s needs [2]. Effective family-centered care can also help address any ethical dilemmas that may arise.

Developmentally Appropriate Care

Neonatal palliative care takes into account the unique needs and developmental stage of the neonate. It aims to optimize their developmental potential and provide appropriate stimulation and interaction. Sensory-based interventions, such as gentle touch, kangaroo care, and music therapy, may be utilized to promote bonding, comfort, and well-being [11,12].

Shared decision-making

Serious illnesses in neonates often present complex medical decisions. By integrating neonatal palliative care into the Greek national healthcare system, healthcare professionals can work collaboratively to ensure seamless transitions between different stages of care, minimizing fragmentation and providing consistent support for families [1,5].

Continuity of care

Integrating neonatal palliative care into the Greek national healthcare system ensures a consecutive continuity of care for neonates and their families. From the time of diagnosis, throughout the neonatal period, and beyond, families can access palliative care services and benefit from them, since they are aligned with other medical treatments and interventions. By encouraging collaboration among various healthcare professionals, gaps in care are minimized and consistent support is ensured. Neonatal palliative care teams warrant coordination of care plans, including discussion about goals of care, treatment decisions, and transitions between healthcare settings [5,13].

Bereavement support

Neonatal palliative care recognizes the profound impact of a neonate’s death on the family. It offers bereavement support to parents, siblings, and the rest family both during the neonate’s life and after its passing. This may involve counseling, support groups, memorial services, and assistance with practical matters related to grief and loss [14].

Education and training

Integrating neonatal palliative care in healthcare systems requires advanced training and education for healthcare professionals. Physicians, midwives, nurses, psychologists, and other professionals often complete continuing education and professional development programs that promote patient- and family-centered services [2,5,15]. Although relevant training programs differ from one European country to another, there are fundamental principles that should underpin all training programs in palliative care. Opportunities for specialized training in neonatal palliative care can be provided by a country's schools of health and care sciences, at an undergraduate and postgraduate level. Specialization in neonatal palliative care can be provided to pediatricians, neonatologists, midwives, nurses, psychologists, physiotherapists, and social workers. It should be noted for Greece that children's palliative care education is limited to a few hours in the aforementioned schools [5]. Courses are either compulsory or elective and focus mainly on theory, as clinical practice is constrained by the existence of few facilities. An exception is an interdisciplinary postgraduate program [5]. Healthcare professionals need advanced and evidence-based training to become an integrated part of their undergraduate, postgraduate, and continuing education programs because education equips healthcare professionals with the skills and knowledge they need to provide compassionate and effective care to neonates and their families [1,15].

Ethical and legal considerations

Neonatal palliative care involves complex ethical and legal considerations, including issues related to decision-making about treatment and withdrawal of care, end-of-life care, bereavement support, and parental autonomy. Integrating these services into the healthcare system enables the establishment of ethical guidelines, protocols, and legislative frameworks that ensure that the best interests of neonates and their families are prioritized [16]. By incorporating neonatal palliative care into the Greek national healthcare system, end-of-life decision-making informed consent, and the reporting of certain conditions or circumstances, depending on the jurisdiction, will be strictly adhered to confidentiality, privacy, and laws, thus promoting a respectful and evidence-based approach.

International standards and best practices

An integrated healthcare system should include palliative care initiatives so as to deliver high-quality, holistic, and culturally responsive palliative care throughout it. International standards and best practices in neonatal care are continually evolving based on scientific research and consensus among medical experts [2,3,5]. Although specific practices may vary across countries due to local resources, infrastructure, and cultural factors, Greece can be attuned to international standards and evidence-based practices, benefiting from the knowledge and experience gained by other countries in this field. Collaboration and sharing of expertise can further improve the quality of neonatal palliative care in Greece.

Conclusions

To successfully integrate neonatal palliative care into the Greek national healthcare system, collaboration among healthcare professionals, policymakers, and relevant stakeholders is vital. The development of guidelines, protocols, and specialized training programs, along with increased public awareness and support, can contribute to the effective implementation of these services. Ultimately, integrating neonatal palliative care will help ensure that every neonate and their family receive the care, support, and dignity they deserve in the face of life-threatening and life-limiting conditions.
